# Immediate Full-Arch Maxillary Rehabilitation Supported by Four Implants: A Retrospective Study with 15 to 20 Years of Follow-Up

**DOI:** 10.3390/jcm15020446

**Published:** 2026-01-06

**Authors:** Miguel de Araújo Nobre, Armando Lopes, Ana Ferro, Carlos Moura Guedes, Ricardo Almeida, Mariana Nunes, Miguel Gouveia, Diogo Santos, Inês Vitor

**Affiliations:** 1 Research, Development and Education Department, MALO CLINIC, Avenida dos Combatentes, 43, Level 11, 1600-042 Lisboa, Portugal; ivitor@maloclinics.com; 2 Oral Surgery Department, MALO CLINIC, Avenida dos Combatentes, 43, Level 9, 1600-042 Lisboa, Portugal; alopes@maloclinics.com (A.L.); aferro@maloclinics.com (A.F.); mnunes@maloclinics.com (M.N.); mgouveia@maloclinics.com (M.G.); dsantos@maloclinics.com (D.S.); 3 Prosthodontic Department, MALO CLINIC, Avenida dos Combatentes, 43, Level 10, 1600-042 Lisboa, Portugal; cmguedes@maloclinics.com (C.M.G.); ralmeida@maloclinics.com (R.A.)

**Keywords:** dental implants, edentulous maxilla, all-on-4, immediate function, implant survival, long term outcome, tilted implants

## Abstract

**Background/Objectives**: Edentulism represents a major public health challenge, causing disorders of social, psychological and biological origin. Full-arch implant-supported restorations represent a viable alternative to mitigate this problem. This study aimed to evaluate immediate implant-supported restorations for the rehabilitation of the edentulous maxilla using four implants and distal implant tilting between 15 and 20 years. **Methods**: A total of 740 patients were included (women: 440; men: 300; average age: 55.3 years) rehabilitated with 740 prostheses, supported by 2960 dental implants. The primary outcome measure was prosthetic/implant cumulative survival and success (CSurR;CSucR). Secondary outcome measures included marginal bone loss (MBL), and the incidence of complications was evaluated as a secondary outcome measure. The outcomes were evaluated at 15 and 20 years. **Results**: In total, 287 patients (38.8%) with 1148 implants (38.8%) were lost to follow-up. A total of 170 implants (5.7%) in 101 patients (13.6%) failed, resulting in an implant CSurR and CSucR of 90.7% and 84.6%, respectively, after up to 20 years of follow-up. The prosthetic success rate was 98.1%. The average MBL was 1.07 mm ± 1.38 mm and 1.46 mm ± 1.56 mm at 15- and 20-years, respectively. Mechanical complication incidence was 78.5%, occurring in 581 patients (provisional prostheses: n = 448, 60.5%; definitive prostheses: *n* = 374, 50.5%). Biological complications occurred in 449 implants (15.2%) in 260 patients (35.1%). Biological complications and smoking habits were major risk indicators. **Conclusions**: Considering the study limitations, it can be concluded that the current rehabilitation concept is a viable treatment option in the long term, with mechanical and biological maintenance being necessary throughout the patients’ lives.

## 1. Introduction

Edentulism is a major problem for oral health, causing biological, psychological and social disorders [1]. The estimated global average prevalence of edentulism in 2019 was almost 7%, with more than 350 million cases worldwide. Among the WHO regions, the highest rate is estimated for the European Region (31.3%), with about 88 million cases occurring in people aged 20 years or more [2,3]. Considering the prevalence and burden of edentulism, implant-supported fixed prostheses emerge as the only reliable solution for the resolution and maintenance of function in the long term.

Immediate full-arch maxillary fixed prosthetic rehabilitations supported by four implants (two axial anterior implants and two tilted posterior implants), as in the All-on-4 concept (Nobel Biocare AB, Gothenburg, Sweden), consists of a treatment alternative to rehabilitate edentulous patients. Based on the insertion of two anterior implants in an axial position and two posterior implants tilted distally, following the anterior wall of the maxillary sinus, it is possible to provide support and function for a full-arch prosthesis immediately, on the same day, while avoiding more complex, time consuming and invasive procedures such as bone grafting [4,5]. This is of particular importance for maxillary rehabilitations, where often low bone density and quantity represent a common finding, and where this immediate full-arch concept provided good outcomes during short-, medium-, and long-term follow-up [6,7]. The possibility of using tilted implants in distal areas provided good anchorage in areas of poor bone quality and quantity and enabled both good anterior–posterior spread and cantilever reduction for increased prosthetic load balance and reduced mechanical stress in peri-implant bone [8,9]. Furthermore, previous systematic reviews registered good outcomes for immediate implant-supported restorations for the edentulous rehabilitation of the maxilla using four implants and distal implant tilting in the two posterior implants [6,10,11]. In a previous study, high cumulative survival rates were reported for implants (94.7%) and prostheses (99.2%), together with an incidence of biological and mechanical complications of 7.8% and 58.8%, respectively, with up to 13 years of follow-up [7]. Nevertheless, studies evaluating the very long-term outcome of full-arch maxillary restorations supported by implants in immediate function are scarce, limiting the validity of this treatment alternative. This study aimed was to investigate the outcome between 15 and 20 years of immediate implant-supported rehabilitations using four implants and posterior implant tilting.

## 2. Materials and Methods

This article was written following the STROBE guidelines for observational studies [12]. This retrospective study was performed at a private rehabilitation center (Maló Clinic, Lisbon, Portugal) and approved by an independent ethics committee (Ethical Committee for Health; Authorization nº 002/2025). The present study was performed in agreement with the Declaration of Helsinki of 1964, as revised in 2013. Patient treatment occurred between November 2002 and December 2008. The inclusion criteria comprised patients requiring fixed full-arch maxillary prosthetic rehabilitation supported by implants in immediate function, due to edentulism or the presence of hopeless teeth, as well as patients with immediate full-arch rehabilitations using four implants and posterior implant treatment for the rehabilitation of the edentulous maxilla, who completed the rehabilitation process at the clinic. Exclusion criteria for performing the rehabilitations included patients in active chemotherapy or radiotherapy, existence of posterior teeth with a healthy status and sufficient bone height allowing implant insertion in the posterior section for partial restorations. The mere presence of a systemic condition or smoking habit was not considered as an exclusion criterion.

For each patient, medical history was assessed, followed by clinical examinations and radiographic assessments (orthopantomography and computerized tomography scan). All procedures were performed under local anesthesia with mepivacaine hydrochloride and epinephrine 1:100,000 (Scandinibsa 2%; Inibsa Laboratory, Barcelona, Spain). Patients received preoperative diazepam (Valium^®^ 10 mg, Roche, Amadora, Portugal) and antibiotic prophylaxis (amoxicillin/clavulanic acid 875/125 mg, Labesfal, Campo de Besteiros, Portugal) starting 1 h before surgery and continued for 6 days postoperatively. A tapering corticosteroid regimen with prednisone (15–5 mg/day, Meticorten®, Schering-Plough Farma, Lda, Agualva-Cacém, Portugal) was prescribed for 4 days, followed by ibuprofen (600 mg, Ratiopharm, Lda, Carnaxide, Portugal) from postoperative days 4 to 7. Analgesics (clonixine 300 mg, Clonix®, Janssen-Cilag Farmaceutica, Lda, Barcarena, Portugal) were administered on the day of surgery and as needed for up to 3 days, and omeprazole (20 mg, Alter SA, Lisbon, Portugal) was prescribed for gastric protection until postoperative day 6. When indicated, teeth were extracted intraoperatively prior to implant placement. A mucoperiosteal flap was then elevated along the ridge crest, with releasing incisions on the buccal aspect of the molar region. A small lateral window was created using a round bur to identify the anterior wall of the maxillary sinus. Implant placement followed standard protocols, with intentional under-preparation of the osteotomies to achieve insertion torque values between 30 and 50 Ncm. Countersinking was performed when necessary to accommodate the implant head of tilted implants and/or to ensure engagement of both buccal and lingual cortical bone in cases of thin crests. The vertical position of the implant platform varied according to implant design: 0.8 mm supracrestal for Mk III/Mk IV implants (Brånemark System; Nobel Biocare AB) and equicrestal for NobelSpeedy implants (Nobel Biocare AB). Bicortical anchorage was achieved whenever possible. The two posterior implants were placed first, tilted 30–45° relative to the occlusal plane and positioned along the anterior wall of the maxillary sinus using an edentulous surgical guide emerging between the first premolar and first molar sites, depending on the degree of maxillary atrophy. Subsequently, two anterior implants were inserted axially following the anatomy of the anterior maxilla. Angulated 30° multi-unit abutments (Nobel Biocare AB) were connected to the posterior implants, while straight (0°) or 17° angulated multi-unit abutments (Nobel Biocare AB) were placed on the anterior implants. Soft tissues were sutured back in position using nonresorbable sutures (3-0, Silkam; B. Braun Aesculap, Center Valley, PA, USA), and abutments were accessed using a mechanical soft-tissue punch (Nobel Biocare AB).

A provisional prosthesis of high-density acrylic resin (PalaXpress Ultra; Heraeus Kulzer GmbH, Hanau, Germany) with acrylic resin teeth (Premium Teeth; Heraeus Kulzer GmbH) and Temporary Multi-unit Titanium Copings (Nobel Biocare AB) was delivered on the same day of surgery. Definitive prostheses were delivered no earlier than 6 months postoperatively and were fabricated according to patient preference, consisting of a titanium framework (Procera; Nobel Biocare AB) veneered with alumina ceramic crowns (Procera Crowns; NobelRondo Ceramics, Nobel Biocare AB) or acrylic resin teeth and high-density acrylic resin.

**Figure 1:**
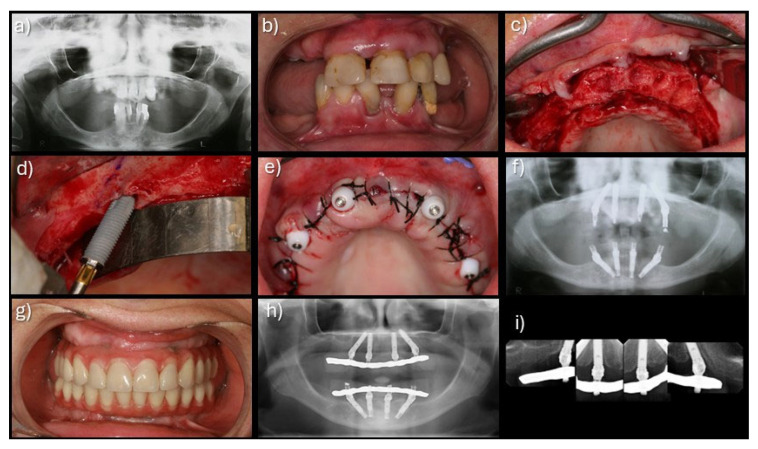
Clinical case of an immediate implant-supported full-arch maxillary rehabilitation using 4 implants and posterior implant tilting, from implant insertion to 20 years: (**a**) pre-treatment orthopantomography; (**b**) pre-treatment intra-oral view; (**c**) per-operative intra-oral view after teeth extraction and bone regularization; (**d**) per-operative intra-oral view of the insertion of the tilted posterior implant anterior to the maxillary sinus; (**e**) per-operative intra-oral view after implant insertion and suturing; (**f**) orthopantomography on the day of surgery with immediate prosthesis in place achieving immediate function; (**g**) intra-oral view of the definitive prostheses in place; (**h**) orthopantomography with the definitive prostheses in place; (**i**) periapical radiographs of the maxillary rehabilitation at 20 years of follow-up.

Patients were instructed to follow a soft-food diet during the initial postoperative period and were enrolled in a standardized maintenance program including oral hygiene instructions [13]. The recall regimen was set at 10 days; 2, 4, and 6 months; 1 year; and every 6 months thereafter, and included prosthesis removal, diagnosis, professional prophylaxis, and reinforcement of oral hygiene measures. A clinical case with very long-term follow-up is illustrated in Figure 1.

Primary outcome measures included prosthetic success and implant survival/success. Prosthetic success was defined as continued function without replacement. The implants were assessed considering success criteria [14] requiring that implants (a) supported the prosthesis without “sleeping”; (b) were stable on manual testing; (c) demonstrated absence of persistent infection that could jeopardize the implant outcome; (d) showed no radiolucency; (e) achieved good aesthetics with no complaints from the prosthodontist nor patient; and (f) allowed a fixed prosthesis that was comfortable and hygienic for the patient. Implants not complying with the criteria were considered survivals. Implant removal was classified as failure. Secondary outcomes included marginal bone loss (MBL) as well as the incidence of mechanical and biological complications. MBL was assessed using periapical radiographs obtained at implant placement and at 15 and 20 years acquired with the parallel technique and a film holder (Super-bite; Hawe Neos, Bioggio, Switzerland), manually adjusted to approximate an orthogonal orientation. The assessment was performed by a calibrated, blinded assessor using image analysis software (iRYS, version 13.0; MyRay, Bologna, Italy). The implant platform (horizontal interface between implant and abutment) served as the reference point, and MBL was defined as the change in marginal bone level relative to surgery. Radiographs were included for analysis only if implant threads were clearly visible, ensuring both image sharpness and proper beam orientation. Measurements were calibrated based on the known distance between implant threads.

Mechanical complications evaluated in this study were fracture or loosening of any prosthetic components, while biological complications assessed included infections, chronic peri-implant disease (pockets over 5 mm, bleeding upon probing, and concurrent presence of marginal bone loss and clinical attachment loss), fistula or abscess formation.

Descriptive statistics were calculated for prosthetic survival (with the prosthetic restoration as the unit of analysis), implant survival/success (with the implant as the unit of analysis), and marginal bone loss (implant as analytical unit). Life tables were used to estimate the cumulative survival rates of prosthesis and implant.

Inferential analyses were conducted to assess potential differences and to identify risk indicators. Multivariate regression models were used for this purpose. Cox proportional hazards models were applied to estimate crude hazard ratios (HRs) with 95% confidence intervals (CIs) for factors associated with the outcome variable “implant failure.” Binary logistic regression models were used to estimate crude odds ratios (ORs) with 95% CIs for factors associated with the outcomes “marginal bone loss > 3 mm,” “biological complications,” and “mechanical complications.” All analyses considered the patient as the unit of analysis.

The analytical strategy consisted of initial univariate analyses to identify covariates associated with each dependent variable. For implant failure, the following variables were evaluated: age, sex, presence of systemic conditions, smoking status, type of opposing dentition (natural teeth, fixed prosthesis on natural teeth, implant-supported prosthesis, removable prosthesis, or miscellaneous), prosthetic material (acrylic resin, metal–acrylic resin, metal–ceramic), cantilever length (none, 1-unit, or 2-unit), and the presence of mechanical or biological complications. For the outcomes “marginal bone loss > 3 mm,” “mechanical complications,” and “biological complications,” the same variables were analyzed, with the addition of previous failure of an adjacent implant. Covariates showing a *p*-value < 0.150 in the univariate analyses were subsequently included in the multivariate models [15]. Additional statistical analyses was performed to evaluate the potential effect and impact of those lost to follow-up on the results. A sensitivity analysis was performed for implant survival estimating the best case (all patients lost to follow-up retained their implants in function) and worst case (all patients lost to follow-up lost all implants) scenarios. A comparison between patients lost to follow-up and patients with completed follow-up was performed for the variables of age (Mann–Whitney U test), gender (Chi-square test), systemic conditions (Chi-square test), and smoking habits (Chi-square test). The level of significance was set at 5%. The IBM SPSS Statistics software version 26 (IBM, Armonk, NY, USA) was used for statistical analysis.

## 3. Results

### 3.1. Sample

This study included 740 patients (440 women and 300 men), with an average age of 55.3 years (ranging between 20 and 88 years).

There were 286 patients presenting conditions considering the International Classification of Disease, version 11 (ICD-11); of these, 216 patients (29.2%) presented a single systemic condition, 70 patients (9.5%) > one condition and 222 patients were smokers (30%) (Table 1). One hundred and ninety-seven patients were judged to have bruxism (26.6%). Two hundred and eighty-seven patients (38.8%) with 1148 implants (38.8%) were lost to follow-up. Forty-two patients (168 implants) were deceased (non-related to the implant treatment), while two hundred and forty-six patients (984 implants) became unreachable. The comparison in baseline characteristics between patients lost to follow-up and patients with completed follow-up yielded a significant difference in age (*p* < 0.001, Mann–Whitney U test), with patients lost to follow-up being older. No significant differences between these two groups were registered for gender (*p* = 0.080, Chi-square test), systemic condition (*p* = 0.432, Chi-square test), and smoking (*p* = 0.742, Chi-square test).

### 3.2. Prostheses and Implants

A total of 740 restorations were attached in the maxilla, supported by 2960 implants—17 Brånemark System Mk III implants [n = 2 failures], 92 Brånemark System Mk IV implants [n = 11 failures], 2851 NobelSpeedy Groovy implants [n = 157 failures]—with widths ranging between 3.3 and 5 mm and lengths ranging from 8.5 to 18 mm and all with anodically oxidized surface. Concerning the opposing dentition, 255 patients presented implant-supported fixed prosthesis, 145 patients presented natural teeth, 315 patients presented a miscellaneous combination of implant-supported fixed prosthesis and natural teeth, 1 patient presented fixed prosthetics over natural teeth, and 24 patients had a removable prosthesis.

### 3.3. Prosthetic Success, Implant Survival/Success and Risk Indicators for Implant Failure

Thirteen patients (1.8%) lost their prostheses: nine patients (1.2%) lost their prostheses due to implant failures, and four patients (0.5%) fractured the prosthesis (one provisional prostheses and three definitive prostheses), rendering a 98.1% prosthetic success rate after up to 20 years of follow-up (Table 2).

A total of 170 implants (5.7%) in 101 patients (52 women and 49 men; 13.6%) failed, with 140 implants lost in the first 180 months and 30 implants lost between 181 months and 240 months, resulting in an implant cumulative survival rate of 94.1% and 90.7% at 15 and 20 years, respectively (Table 3). Implant failures occurred in 50 patients with smoking habits, in 31 patients with bruxism and in 12 patients with both conditions. The sensitivity analysis estimated a potential cumulative implant survival rate at 20 years of 93.1% and 59.4% for best and worst case scenarios, respectively (Table 4). From the patients with implant failures, 83 patients lost implants, with the prostheses kept in function and supported on the remaining implants for a healing period of 6 months, followed by the insertion of new implants (not accounted in this study) to restore function supported on 4 implants; 18 patients lost implants and refused surgery, opting to have their prostheses supported on the remaining implants; and 9 patients lost implants and prostheses. Adding to the 170 failed implants, 127 implants were judged as unsuccessful after applying the success criteria, registering a cumulative implant success rate of 89.4% and 84.6% at 15 and 20 years, respectively (Table 5).

Univariately, the regression analysis registered associations between implant failure and gender (*p* = 0.058), systemic condition (*p* = 0.053), smoking (*p* < 0.001), opposing dentition (*p* = 0.119), and biological complications (*p* < 0.001) (Cox proportional hazards). The multivariate analysis disclosed the variables smoking (HR = 1.93) and biological complications (HR = 2.18) as risk indicators for implant failure, when controlled for sex, presence of systemic conditions and opposing dentition (Cox proportional hazards, Table 6).

### 3.4. Marginal Bone Loss and Risk Indicators for MBL > 3 mm

**Figure 2:**
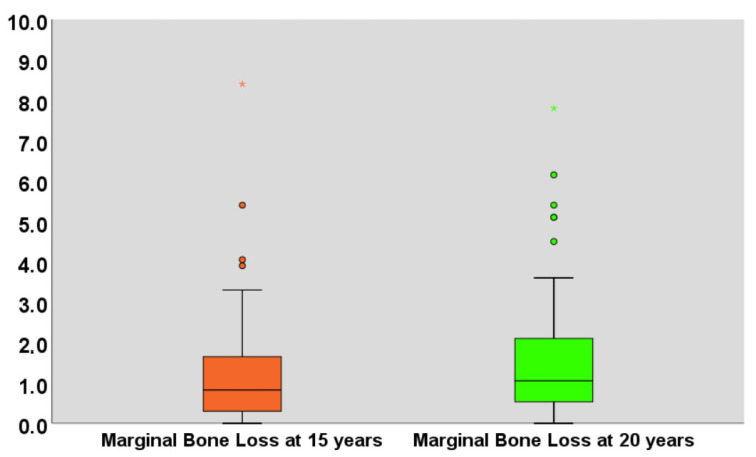
Boxplot illustrating the marginal bone loss measured in millimeters at 15 and 20 years of follow-up. The median is represented by the horizontal black line inside the box; the lower box edge represents the 25th percentile; the upper box edge represents the 75th percentile; whiskers represent the standard deviation; asterisk and dots represent outlier values.

In total, 74% and 93% of the implant radiographs were readable for marginal bone loss at 15 and 20 years, respectively. At 15 and 20 years follow-up, the average (standard deviation) marginal bone loss was 1.07 mm ± 1.38 mm, and 1.46 mm ± 1.56 mm (Figure 2, Table 7).

There were 63 patients with 95 implants and 8 patients with 14 implants exceeding MBL of 3 mm at 15 years and 20 years, respectively. The univariate logistic regression analysis registered smoking (*p* < 0.001), mechanical complications (*p* = 0.031), biological complications (*p* = 0.002) and previous failure of a contiguous implant (*p* = 0.021) as potentially associated with MBL > 3 mm. In the multivariate analysis, the variables smoking (OR = 3.60) and biological complications (OR = 3.70) were considered as risk indicators for MBL, while mechanical complications had a protective effect (OR = 0.26), when controlled for the presence of a previous failure of a contiguous implant (Table 8).

### 3.5. Mechanical Complications and Incidence Risk Indicators

Mechanical complications were observed in 581 patients (78.5%): provisional prostheses (n = 448 patients; 60.5%) and definitive prostheses (n = 374 patients; 50.5%) (Table 9), for a total of 144 patients accumulating complications in both prostheses. Of these complications, 299 occurred in patients diagnosed with bruxism (n = 186), and 301 occurred in patients whose prostheses occluded against implant-supported fixed prostheses in the opposing dentition (n = 200). Occlusal adjustments were performed for all patients, loose components were retightened, and provisional prostheses were repaired. Minor ceramic chippings were adjusted, and major ceramic chipping or fracture implied the replacement of the crown; nightguards were manufactured and placed in patients judged to be bruxers. No significant associations between potential risk indicators and mechanical complications were found (Table 10).

### 3.6. Biological Complications and Incidence Risk Indicators

Biological complications occurred in 449 of the implants (15.2%) in 260 patients (35.1%). Implant infections during the first year of follow-up were recorded in 117 implants across 81 patients, leading to 7 implant failures. Chronic peri-implant disease affected 332 implants in 199 patients and resulted in 54 implant failures. In 175 patients with 249 implants, the problem was solved (non-surgically: 222 implants in 164 patients; surgically: 27 implants in 23 patients). For 200 implants in 123 patients, the problem was not resolved after non-surgical and surgical treatment, resulting in 61 implant losses and 139 implants considered as survivals, 6 implants (4 patients) that were lost to follow-up immediately after diagnosis, and 7 implants in 6 patients whose treatment outcome was not yet confirmed at the time of study completion. The univariate analysis disclosed age (*p* < 0.001), smoking (*p* < 0.001) and opposing dentition (miscellaneous: *p* = 0.084) as variables potentially associated with the incidence of biological complications. In the multivariate analysis, age (OR = 0.48) and smoking (OR = 1.78) remained significant when controlled for the presence of opposing dentition (Table 11).

## 4. Discussion

The present study, to the authors knowledge, registered the longest follow-up time (up to 20 years) for immediate full-arch implant-supported maxillary rehabilitations using four implants (two posterior implants tilted distally and two axially oriented anterior implants). Our study reported good outcomes for implants placed in immediate function, registering a 98.1% prosthetic success rate, 90.7% cumulative implant survival and 84.6% cumulative implant success rates. This study extends the results obtained in previous publications including a sample of 1072 patients with a 13-year 99.2% prosthetic success rate and a 94.7% cumulative implant survival rate [7], as well as previous meta-analytic studies [6,10], further validating the current full-arch concept. In the absence of studies applying the immediate function approach to full-arch maxillary rehabilitations in very long-term follow-up, the authors compare the results with other studies using two-stage surgical approaches and very long-term follow-up. Previous studies [16,17,18] with follow-ups between 20 and 25 years registered an implant survival rate ranging between 71.05% and 99.2%. The implant survival rate of the present study is within the range reported in these previous publications [16,17,18], despite the more comprehensive inclusion criteria (including patients with history of periodontitis, unlike Carossa et al. [18], the significantly greater sample size and follow-up (compared to Astrand et al. [16]), and the higher survival rate despite the smaller sample size and follow-up (compared to Jemt et al. [17])).

The analysis of survival provides a good overview for long-term success while attesting to the non-superiority of two-stage protocols over immediate loading. Moreover, it does so underlining the stability that it is possible to maintain using only four implants for support of a full-arch maxillary restoration, even in the presence of implant failures. However, it is also important to underline that implant failures and complications occur frequently in very long-term follow-ups. In the present study, 170 implant failures occurred in 101 patients (with 67 implant failures in 36 patients during the first year of function). The process of resolving an implant failure and restoring full prosthetic support through the insertion of implants (as in the case of the present study) is burdensome to both patient and clinician, with physical and monetary costs that need to be considered and informed at the pre-treatment phase, stressing the need for structured surgical, prosthodontic and maintenance protocols. Nevertheless, the trend for a higher implant failure rate during the biological osseointegration period was previously documented, with a pattern of failure for implants from the same system observed during healing or the first year of function [19].

The Cox proportional hazards regression and the binary logistic regression models determined the variables biological complications and smoking habits as risk indicators for both implant failure and marginal bone loss exceeding 3 mm (with smoking also significantly associated with biological complications). Biological complications have long been associated with implant failure, particularly with late implant failure, with a significant number of studies providing evidence of its harmful effect [20,21,22,23,24,25]. With infection indicated as a significant factor for implant failures [20,21], chronic peri-implant disease is considered the attributable cause for late failures [22,25], as in most situations registered in the present study. Moreover, it is plausible to assume biological complications as a risk indicator for increased marginal bone loss, since the destruction of the peri-implant complex is the means of disease expression, resulting in marginal bone loss. The clinical significance of this result relies on the need for primary prevention (good oral hygiene and regular professional care) in order to prevent the disease [24], and secondary prevention (to address the complication through non-surgical and surgical approaches as soon as possible) [23]. Smoking aggregates a significant number of publications that previously attested to its deleterious effect on the successful outcome of implant-supported rehabilitations, with a previous systematic review and meta-analysis indicating almost three times increased odds for implant failure in smokers compared to non-smokers [26]. Furthermore, smoking has the potential to significantly impact both early and late failures by negatively influencing the osseointegration process, impairing the immune response [27], and most notably, providing a chronic inflammation state that increases the probability of biological complications occurring [28], acting as a facilitating factor. In our study, smoking was the only variable present in three models, registered as a risk indicator for implant failure, biological complications and increased marginal bone loss simultaneously. This result is of paramount clinical significance, deeming it necessary to inform patients about the risks that smoking habits may introduce in the outcome of their implant-supported rehabilitations prior to performing the treatment, and referring the patient for smoking cessation appointments.

The fact that the incidence of mechanical complications was registered as a protective effect for marginal bone loss in our sample may be explained clinically and epidemiologically. On one hand, the mechanical complication acts as a warning sign and is the weakest link that occurs in lieu of a more serious event, such as a loss of osseointegration or a substantial marginal bone loss around the implant. On the other hand, patients with mechanical complications needed to be present in more clinical appointments, in which the prostheses were removed and prophylaxis was performed, consisting of the probable real protective factor for marginal bone loss [29], acting as a “second intention pseudo-maintenance protocol”. Nevertheless the average marginal bone loss registered in the present study represented a stable trend, building from the results of previous publications by our group on the same concept and using patients from the current sample, with averages of 0.9–1.2 mm at 1 year [4,30], 1.18 mm at 5 years [7], and 1.67 mm at 10 years [7]. From a clinical point of view, it should be noted that marginal bone loss may be influenced by complications occurring during osseointegration or by chronic conditions (including the effect of smoking or peri-implant disease) in the long term, requiring its assessment during regular follow-ups.

The 78.5% cumulative incidence rate for mechanical complications at the patient level (with 60.5% and 50.5% in provisional and definitive prostheses, respectively) represents a consequence of the very long-term exposure to function, imposing substantial prosthetic maintenance over time, as previously reported in a 20-year follow-up study in edentulous patients with implant-supported fixed prostheses [31]. Attard et al. [31] noted that prosthetic maintenance was ongoing, including fractured components and replacement of prostheses, and that the longevity of a fixed prosthesis for this group of patients was on average 8.39 ± 5.30 years. Clinically, it poses the need to inform the patient beforehand that maintenance or replacement of prosthetic components (and consequent costs to cover material and clinical appointments) is going to be needed throughout the patients’ life.

The cumulative incidence of biological complications was 15.2% at implant level, affecting 35.1% of patients (n = 260 patients). Previous systematic reviews estimated the occurrence of biological complications in a range between 19.5% and 85% [32,33], a very large confidence interval that is related to not only different diagnostic criteria but also different exposure times. Epidemiologically, it is necessary to take into consideration that increased follow-up times are linked to increased exposure times to risk factors, be they environmentally related (such as smoking) or host-related (such as the patients’ age), precisely the two variables that were significantly associated with an increased probability of biological complications. Nevertheless, both associations between smoking and age with chronic peri-implant disease, from an epidemiological point of view, should be attributed to a confounding effect. Smoking was described as a confounder in the association with chronic peri-implant disease in previous studies [34,35], with smoking strongly associated with periodontitis [34], and therefore, with a history of periodontitis as the underlying risk indicator for chronic peri-implant disease, as stated in previous systematic reviews [36,37]. The effect registered for age, with patients over 60 years at decreased risk for biological complications, can be explained by the increased time of exposure, as younger patients at the time of surgery are more likely to be exposed for a longer period of time during function compared to older patients. In addition, in our sample, patients under 60 years of age had an 18% increased prevalence of smoking habits compared to patients ≥ 60 years of age (40.7% vs. 22.8% smoking prevalence, respectively). It should be noted that the multivariable analysis lacked the inclusion of variables such as the history of periodontitis and bacterial plaque, representing a limitation of the present study. From a clinical perspective, the impact of biological complications makes it mandatory, first, to inform the patient beforehand that prevention is key and, second, that chronic peri-implant disease may require additional interventions for its resolution, including surgical procedures and associated costs.

The study strengths include the large sample size and multivariate statistical analysis applied to the different outcome measures. The large sample size enabled a higher statistical power and better representation of the population, reducing the impact of outliers and ensuring higher reliability. The multivariate analysis allowed a holistic view of the data and greater statistical efficiency, enhancing both accuracy and prediction. This in turn enables better forecasting from a clinical point of view, analyzing multiple factors that influence the outcome risk, which is of clinical and practical relevance.

The limitations of the present study include the study being single-centered, reducing external validity; the retrospective design, which may result in unmeasured or missing data and marginal bone loss being underestimated considering the 26% and 7% ineligible radiographs at 15 and 20 years, respectively; and being performed by a single outcome assessor. A further limitation was those lost to follow-up reaching 38.8%. The potential impact of this limitation on the outcome was illustrated by sensitivity analysis, where a 33.7% difference between the best case and worst case scenarios was estimated. A best case scenario of 93.1% and a worst case scenario of 59.4% in cumulative implant survival rate were estimated, warranting caution in the evaluation of the results due to the wide range in potential survival. A comparison between patients lost to follow-up and those with complete data was performed for age, sex, systemic conditions, and smoking. This analysis yielded a significant difference only for age, with patients lost to follow-up being older, posing an increased risk of selection bias. However, it is likely that an underestimation of deceased patients occurred, taking into consideration the average age at the time of surgery (55 years) and the very long-term follow-up with up to 20 years. Moreover, the present study’s follow-up crossed the COVID-19 pandemic, where age was the most significant factor for mortality, with odds increasing 20 times for the age group our sample would be at after 20 years of follow-up (71 to 75 years of age) [38]. Taking these points into account, a high loss to follow-up rate should be anticipated, similar to previous long-term publications, with over 56% reporting lost to follow-up rates [16,17]. Considering the study limitations, the interpretation of the results warrants caution as it is susceptible to overestimation of survival and underestimation of both complications and marginal bone loss. Future studies should prioritize the reporting of very long-term outcomes of immediate full-arch maxillary rehabilitation with four-implant-supported fixed prostheses, incorporating different restorative materials and implant micro designs to substantiate and compare alternatives to the current treatment protocol, which stands as the gold standard for this concept.

## 5. Conclusions

Based on the results, the high implant and prosthetic survival rates at 20 years and the low marginal bone loss demonstrate that full-arch fixed prosthetic maxillary rehabilitations supported by four implants (two posterior implants tilted distally and two anterior implants axially oriented) is a viable treatment option in the long term. Nevertheless, implant failures occur (the majority during the biological osseointegration period), as well as biological and mechanical complications during long-term follow-up; however, the existence of surgical, prosthetic and maintenance protocols is viewed as necessary for maintaining success in the long term, given that the maintenance and resolution of mechanical and biological complications are going to be needed throughout the patients’ life. Patients susceptible to biological complications and with smoking habits warrant special vigilance, considering the deleterious influence of these two factors on implant survival, marginal bone loss and biological complications.

## Figures and Tables

**Table 1:** Overall medical status according to the ICD-11, including the distribution of patients deceased and lost to follow-up in the sample.

ICD-11 Classification	ICD-11 Group Description	Examples	Number of Patients	Number of Implants
1	Certain infectious or parasitic diseases	(HIV, hepatitis)	26	104
2	Neoplasms	(Cancer)	24	96
3	Diseases of the blood or blood forming organs	(Coagulation problems)	4	16
4	Diseases of the immune system	(Lupus erythematosus)	1	4
5	Endocrine, nutritional, or metabolic diseases	(Diabetes, hypercholesterolemia, hyperthyroidism)	71	284
6	Mental, behavioral, or neurodevelopmental disorders	(Depression, bipolar, opioid dependence)	7	28
8	Diseases of the nervous system	(Alzheimer’s, epilepsy, intracerebral hemorrhage)	16	64
9	Diseases of the visual system	(Optic nerve stenosis, glaucoma)	2	8
11	Diseases of the circulatory system	(Hypertension, arrhythmia, angina)	166	664
12	Diseases of the respiratory system	(Emphysema, asthma, sinusitis)	11	44
13	Diseases of the digestive system	(Esophagitis, Crohn disease, gastritis)	7	28
14	Diseases of the skin	(Epidermolysis bullosa, psoriasis)	3	12
15	Diseases of the musculoskeletal system or connective tissue	(Osteoporosis, rheumatoid arthritis)	23	92
16	Diseases of the genitourinary system	(Interstitial cystitis, hyperplasia of prostate, menopause)	4	16
18	Pregnancy, childbirth or the puerperium	(Hysterectomy)	1	4
21	Symptoms, signs, findings	(Hemiplegia, nervous tachycardia)	2	8
24	Factors influencing health status or contact with health services	(Smoking, other specified acquired absence of organ, presence of orthopedic joint implants)	225	900
X	Squamous cell neoplasms	(Papilloma, seminal vesicle, vertebral column)	3	12
Total			596 ^a^	2384
**Distribution of deceased and lost to follow-up patients ^b^**				
First year	1 patient deceased	2 patients unreachable	Total of 3 patients	
1–2 years	2 patients deceased	16 patients unreachable	Total of 18 patients	
2–3 years	1 patient deceased	10 patients unreachable	Total of 11 patients	
3–4 years	1 patient deceased	19 patients unreachable	Total of 20 patients	
4–5 years	1 patient deceased	11 patients unreachable	Total of 12 patients	
5–6 years	5 patients deceased	19 patients unreachable	Total of 24 patients	
6–7 years	4 patients deceased	18 patients unreachable	Total of 22 patients	
7–8 years	5 patients deceased	19 patients unreachable	Total of 24 patients	
8–9 years	3 patients deceased	12 patients unreachable	Total of 15 patients	
9–10 years	1 patient deceased	16 patients unreachable	Total of 17 patients	
10–11 years	2 patients deceased	14 patients unreachable	Total of 16 patients	
11–12 years	1 patient deceased	22 patients unreachable	Total of 23 patients	
12–13 years	2 patients deceased	24 patients unreachable	Total of 26 patients	
13–14 years	2 patients deceased	21 patients unreachable	Total of 23 patients	
14–15 years	4 patients deceased	30 patients unreachable	Total of 34 patients	
15–16 years	1 patient deceased	-	Total of 1 patient	
16–17 years	1 patient deceased	-	Total of 1 patient	
17–18 years	2 patients deceased	-	Total of 2 patients	
18–19 years	2 patients deceased	-	Total of 2 patients	
19–20 years	1 patient deceased	-	Total of 1 patient	

^a^ A total of 216 patients presented a single condition, with 70 patients with more than one condition. ^b^ A total of 287 patients were lost to follow-up. “-” represents out of bound values.

**Table 2:** Life table estimating the prosthetic success rate.

Time	Prostheses		Success Rate (%)	Cumulative Success Rate (%)
	**Total**	**Failed**		
Placement–6 months	740	6	99.2%	99.2%
6 months–1 year	727	3	99.6%	98.8%
1–2 years	705	1	99.9%	98.7%
2–3 years	691	1	99.9%	98.6%
3–4 years	670	0	100%	98.6%
4–5 years	658	0	100%	98.6%
5–6 years	635	1	99.8%	98.4%
6–7 years	613	0	100%	98.4%
7–8 years	589	0	100%	98.4%
8–9 years	575	0	100%	98.4%
9–10 years	558	1	99.8%	98.2%
10–11 years	540	0	100%	98.2%
11–12 years	515	0	100%	98.2%
12–13 years	494	0	100%	98.2%
13–14 years	467	0	100%	98.2%
14–15 years	435	0	100%	98.2%
15–16 years	381	0	100%	98.2%
16–17 years	257	0	100%	98.2%
17–18 years	102	0	100%	98.2%
18–19 years	44	0	100%	98.2%
19–20 years	22	0	100%	98.2%

**Table 3:** Life table estimating the cumulative implant survival rate.

Time	Implants			Survival Rate (%)	Cumulative Survival Rate (%)
	Total	Failed	Lost to Follow-Up		
Placement–6 months	2960	49	4	98.3%	98.3%
6 months–1 year	2907	18	64	99.4%	97.7%
1–2 years	2825	4	48	99.9%	97.6%
2–3 years	2773	5	76	99.8%	97.4%
3–4 years	2692	2	47	99.9%	97.3%
4–5 years	2643	2	88	99.9%	97.3%
5–6 years	2553	3	83	99.9%	97.1%
6–7 years	2467	4	92	99.8%	97.0%
7–8 years	2371	7	53	99.7%	96.7%
8–9 years	2311	8	64	99.6%	96.4%
9–10 years	2239	5	64	99.8%	96.1%
10–11 years	2170	10	99	99.5%	95.7%
11–12 years	2061	5	82	99.8%	95.4%
12–13 years	1974	7	104	99.6%	95.1%
13–14 years	1863	8	126	99.6%	94.7%
14–15 years	1729	10	187	99.4%	94.1%
15–16 years	1532	13	482	99.0%	93.2%
16–17 years	1037	6	597	99.2%	92.4%
17–18 years	434	2	245	99.4%	91.8%
18–19 years	187	2	25	98.9%	90.7%
19–20 years	160	0	160	100%	90.7%

**Table 4:** Sensitivity analysis: life table estimating the cumulative implant survival rate assuming best and worst case scenarios.

Best Case Scenario (Patients Lost to Follow-Up Retain All Implants)					
Time	Implants			Survival Rate (%)	Cumulative Survival Rate (%)
	Total	Failed	Patients Deceased		
Placement–6 months	2960	49	4	98.3%	98.3%
6 months–1 year	2907	18	8	99.4%	97.7%
1–2 years	2881	4	8	99.9%	97.6%
2–3 years	2869	5	4	99.8%	97.4%
3–4 years	2860	2	12	99.9%	97.4%
4–5 years	2846	2	15	99.9%	97.3%
5–6 years	2829	3	19	99.9%	97.2%
6–7 years	2807	4	6	99.9%	97.0%
7–8 years	2797	7	18	99.7%	96.8%
8–9 years	2772	8	1	99.7%	96.5%
9–10 years	2763	5	4	99.8%	96.4%
10–11 years	2754	10	12	99.6%	96.0%
11–12 years	2732	5	15	99.8%	95.8%
12–13 years	2712	7	8	99.7%	95.6%
13–14 years	2697	8	12	99.7%	95.3%
14–15 years	2677	10	199	99.6%	94.9%
15–16 years	2468	13	615	99.4%	94.4%
16–17 years	1840	6	928	99.6%	93.9%
17–18 years	906	2	463	99.7%	93.7%
18–19 years	1441	2	152	99.5%	93.1%
19–20 years	287	0	287	100%	93.1%
**Worst case scenario (patients lost to follow-up lose all implants)**					
**Time**	**Implants**			**Survival Rate (%)**	**Cumulative Survival Rate (%)**
	**Total**	**Failed**	**Patients deceased**		
Placement–6 months	2960	49	4	98.3%	98.3%
6 months–1 year	2907	74	8	97.5%	95.8%
1–2 years	2825	44	8	98.4%	94.3%
2–3 years	2773	77	4	97.2%	91.7%
3–4 years	2692	41	12	98.5%	90.3%
4–5 years	2639	71	15	97.3%	87.9%
5–6 years	2553	71	19	97.2%	85.4%
6–7 years	2463	86	6	96.5%	82.4%
7–8 years	2371	50	18	97.9%	80.7%
8–9 years	2303	63	1	97.3%	78.5%
9–10 years	2239	65	4	97.1%	76.2%
10–11 years	2170	97	12	95.5%	72.8%
11–12 years	2061	80	15	96.1%	70.0%
12–13 years	1966	95	8	95.2%	66.6%
13–14 years	1863	126	12	93.2%	62.1%
14–15 years	1725	10	199	99.4%	61.7%
15–16 years	1516	13	498	99.0%	61.0%
16–17 years	1005	6	577	99.2%	60.5%
17–18 years	422	2	229	99.3%	60.1%
18–19 years	191	2	35	99.8%	59.4%
19–20 years	154	0	154	100%	59.4%

**Table 5:** Life table estimating the cumulative implant success rate.

Time	Implants			Success Rate (%)	Cumulative Success Rate (%)
	Total	Failed	Lost to Follow-Up		
Placement–6 months	2960	49	4	98.3%	98.3%
6 months–1 year	2907	24	64	99.2%	97.5%
1–2 years	2819	11	48	99.6%	97.2%
2–3 years	2760	13	76	99.5%	96.7%
3–4 years	2671	10	51	99.6%	96.3%
4–5 years	2610	11	80	99.6%	95.9%
5–6 years	2519	13	85	99.5%	95.4%
6–7 years	2421	13	88	99.5%	94.9%
7–8 years	2320	14	61	99.4%	94.3%
8–9 years	2245	14	54	99.4%	93.8%
9–10 years	2177	12	62	99.4%	93.2%
10–11 years	2103	14	99	99.3%	92.6%
11–12 years	1990	13	89	99.3%	92.0%
12–13 years	1888	9	92	99.5%	91.6%
13–14 years	1787	17	124	99.0%	90.7%
14–15 years	1646	23	182	98.6%	89.4%
15–16 years	1441	20	472	98.6%	88.2%
16–17 years	949	8	542	99.2%	87.5%
17–18 years	399	6	222	98.5%	86.1%
18–19 years	171	3	26	98.2%	84.6%
19–20 years	142	0	142	100%	84.6%

**Table 6:** Multivariate analysis of potential hazard risk indicators associated with implant failure using the Cox proportional hazards regression model.

Variables	Hazard Ratio Univariate	*p*	Multivariate β	Multivariate Standard Error	*p*	Multivariate Hazard Ratio (95% CI)
Age (≥60 years)	1.00	0.699				
Sex (male)	1.46	0.058	0.33	0.20	0.106	1.39 (0.93–2.07)
Systemic condition	0.66	0.053	−0.36	0.22	0.100	0.70 (0.45–1.07)
Smoking	2.36	<0.001	0.66	0.21	0.001	1.93 (1.29–2.89)
Opposing dentition	0.90	0.119			0.721	
Natural teeth			1.0			
Fixed prostheses over natural teeth			−9.54	258.62	0.971	---
Implant supported prosthesis			−0.11	0.26	0.665	0.89 (0.53–1.49)
Removable prostheses			−0.70	0.74	0.344	0.50 (0.12–2.12)
Miscellaneous			−0.31	0.27	0.240	0.73 (0.43–1.23)
Restorative material	0.89	0.430				
Mechanical complications	1.01	0.973				
Biological complications	2.47	<0.001	0.78	0.21	<0.001	2.18 (1.45–3.27)

“---” represents out of bound values.

**Table 7:** Marginal bone loss registered at 15 and 20 years.

15 Years Follow-Up		
Average (mm)	1.07	
SD (mm)	1.38	
Number (%) of implants with readable radiographs	1137 (74)	
Frequencies	N	%
0 mm	251	22.1
0.1–1 mm	486	42.7
1.1–2 mm	206	18.1
2.1–3 mm	99	8.7
>3 mm	95	8.4
**20 years of follow-up**		
Average (mm)	1.46	
SD (mm)	1.56	
Number (%) of implants with readable radiographs	149 (93)	
Frequencies	N	%
0 mm	15	10.1
0.1–1 mm	55	36.9
1.1–2 mm	41	27.6
2.1–3 mm	24	25.5
>3 mm	14	9.7

**Table 8:** Regression analysis to evaluate the potential risk indicators for the incidence of MBL > 3 mm.

	Univariate Analysis		Multivariate Analysis	
Factor	OR (95% CI)	*p*	OR (95% CI)	*p*
Age (≥60 years)	0.97 (0.92–1.02)	0.203		
Sex (male)	1.47 (0.64–3.36)	0.362		
Systemic conditions	0.65 (0.27–1.56)	0.338		
Smoking	4.67 (2.01–10.88)	<0.001	3.82 (1.58–9.20)	0.003
Opposing dentition	1.04 (0.79–1.38)	0.763		
Restorative material	1.02 (0.55–1.88)	0.951		
Mechanical complications	0.33 (0.12–0.90)	0.031	0.26 (0.09–0.78)	0.016
Biological complications	4.59 (1.78–11.83)	0.002	4.02 (1.49–10.83)	0.006
Previous failure of a contiguous implant	3.06 (1.19–7.90)	0.021		

**Table 9:** Incidence of mechanical complications in provisional and definitive prostheses.

Complications	Number	Percentage
**Provisional prostheses**		
Loosening of prosthetic screws	11	2.5
Loosening of abutment screws	272	60.7
Milled abutments	10	2.2
Abutment screw fractures	5	1.1
Titanium cylinder fractures	32	7.1
Prosthesis fracture	118	26.3
**Definitive prostheses**		
Loosening of prosthetic screws	11	2.9
Loosening of abutment screws	51	13.6
Milled abutments	32	8.6
Abutment screw fractures	5	1.3
Titanium cylinder fractures	7	1.9
Crown fracture	258	69
Prosthesis fracture	9	2.4
Artificial gingiva fracture	1	0.3

**Table 10:** Univariate analysis. Binary logistic regression to evaluate the potential risk indicators for the incidence of mechanical complications.

	Univariate Analysis	
Factor	OR (95% CI)	*p*
Age (≥60 years)	0.99 (0.97–1.01)	0.312
Sex (male)	1.07 (0.75–1.53)	0.707
Systemic conditions	1.03 (0.72–1.48)	0.856
Smoking	0.89 (0.61–1.30)	0.549
Opposing dentition	0.94 (0.84–1.06)	0.317
Restorative material	1.07 (0.82–1.38)	0.626
Biological complications	---	0.997
Previous failure of a contiguous implant	---	0.999

“---” represents out of bound values.

**Table 11:** Binary logistic regression analysis to assess risk indicators for the incidence of biological complications.

	Univariate Analysis		Multivariate Analysis	
Factor	OR (95% CI)	*p*	OR ^a^ (95% CI)	*p*
Age (≥ 60 years)	0.43 (0.30–0.61)	<0.001	0.48 (0.33–0.69)	<0.001
Sex (male)	0.99 (0.73–1.35)	0.949		
Systemic condition	0.92 (0.67–1.25)	0.581		
Smoking	2.05 (1.48–2.83)	<0.001	1.78 (1.27–2.48)	0.001
Opposing dentition		0.468		0.595
Natural teeth	1.0		1.0	
Fixed prostheses over natural teeth	--	1.000	---	1.000
Implant supported prosthesis	0.83 (0.55–1.27)	0.391	0.86 (0.56–1.32)	0.484
Removable prostheses	1.07 (0.45–2.58)	0.877	1.23 (0.50–3.04)	0.656
Miscellaneous	0.70 (0.46–1.05)	0.084	0.74 (0.49–1.13)	0.165
Restorative material	1.08 (0.86–1.35)	0.505		
Mechanical complications	--	0.998		
Previous failure of a contiguous implant	--	0.998		

^a^ OR from logistic regression analysis with age and smoking included given its significance (*p* < 0.150) in the unadjusted model. No esthetic complaints were registered. “--, ---” represents out of bound values.

## Data Availability

The data is available at a public repository link: https://osf.io/dyjr7/overview.
